# Effects of smoking cessation on taste function in heavy smokers undergoing hemiglossectomy for tongue squamous cell carcinoma

**DOI:** 10.1007/s00405-025-09590-8

**Published:** 2025-07-23

**Authors:** Paolo Boscolo-Rizzo, Thomas Hummel, Alberto Vito Marcuzzo, Antonino Maniaci, Giacomo Spinato, Luca Raimondo, Luigi Angelo Vaira, Rachele Tulissi, Anna Menini, Franco Trabalzini, Enzo Emanuelli, Vittorio Grill, Jerry Polesel, Fabiola Giudici, Giancarlo Tirelli

**Affiliations:** 1 Department of Medical, Surgical and Health Sciences, Section of Otolaryngology, University of Trieste, Strada di Fiume 447, Trieste, 34149 Italy; 2 Department of Otorhinolaryngology, Smell & Taste Clinic, Technical University of Dresden, Dresden, Germany; 3 Faculty of Medicine and Surgery, University of Enna “Kore”, Enna, Italy; 4 Department of Neurosciences, Section of Otolaryngology, University of Padova, Treviso, Italy; 5 Unit of Otolaryngology, Humanitas “Gradenigo”, Torino, Italy; 6 Department of Medicine, Surgery and Pharmacy, Maxillofacial Surgery Operative Unit, University of Sassari, Sassari, Italy; 7 Neurobiology Group, SISSA, Scuola Internazionale Superiore di Studi Avanzati, Trieste, Italy; 8 Department of Otorhinolaryngology, Meyer Hospital, I.R.C.C.S, University of Firenze, Firenze, Italy; 9 Department of Life Sciences, University of Trieste, Trieste, Italy; 10 Unit of Cancer Epidemiology, Centro di Riferimento Oncologico di Aviano (CRO) IRCCS, Aviano, Italy

**Keywords:** Taste perception, Oral cavity, Squamous cell carcinoma, Heavy smoking, Gustatory dysfunction, Smoking cessation, Surgery

## Abstract

**Purpose:**

Smokers with squamous cell carcinoma (SCC) of the tongue may be particularly prone to experience multifactorial gustatory dysfunction. This study investigates the effects of heavy smoking on taste perception in patients undergoing glossectomy type IIIa (i.e. non-compartment hemiglossectomy) for SCC of the tongue.

**Methods:**

Gustatory function was assessed in 30 heavy-smoking patients with SCC of the tongue using a validated taste strips test. Psychophysical evaluations were conducted at baseline and at 3, 6, 9, and 12 months post-surgery.

**Results:**

At baseline, mean taste strip scores (TSS [SD]) were significantly lower in patients compared to controls (8.7 [1.9] vs. 11.6 [2.1], *P* <.001). Post-treatment, TSS in patients declined to 7.2 [1.0] at 3 months, followed by a gradual recovery to 7.6 [2.3] at 6 months, 8.4 [2.8] at nine months, and 8.9 [3.9] at 12 months. Patients who quit smoking achieved significant recovery, with TSS improving from 7.4 [0.9] at three months to 11.8 [1.4] at twelve months, nearing control values. In contrast, smokers experienced a progressive decline, from 6.8 [1.2] at three months to 4.6 [1.6] at twelve months (*P* <.001). Adjuvant radiotherapy exacerbated short-term deficits (TSS of 6.6 [1.0] vs. 7.5 [0.9], *P* =.027 at three months) but showed no significant long-term impact.

**Conclusion:**

Persistent smoking worsens taste perception and impedes recovery in patients undergoing glossectomy type IIIa for tongue SCC. Smoking cessation significantly enhances sensory restoration, underscoring its importance in post-treatment care.

**Supplementary Information:**

The online version contains supplementary material available at 10.1007/s00405-025-09590-8.

## Introduction

Taste, a fundamental sensory function for recognizing essential nutrients and avoiding harmful substances, plays a crucial role in maintaining nutritional health and improving quality of life [1]. Squamous cell carcinoma (SCC) of the tongue is the most common malignancy within the oral cavity [2], and its management typically involves surgical excision of the primary tumor and neck dissection followed by pathologic characteristics–guided adjuvant therapy [3].

Gustatory dysfunction is a prevalent complication in patients with SCC of the tongue, resulting from both the pathological mechanisms of the disease and the iatrogenic effects of treatment. SCC of the tongue itself, or the widespread chronic inflammatory state often co-present with it [4], can lead to impaired gustatory perception. Additionally, treatments such as surgical resection, radiotherapy, and chemotherapy may further disrupt the function of taste buds, salivary glands, and neural pathways essential for gustatory processing [5–8].

Globally, the majority of SCCs of the oral cavity are attributable to exposure to tobacco, either through smoking or the use of smokeless tobacco products [9–11]. Heavy smokers are particularly vulnerable to taste impairment, as tobacco directly affects the gustatory system, further exacerbating taste dysfunction in these patients [12, 13]. Consequently, individuals with SCC of the tongue who use tobacco are at an especially high risk of developing severe gustatory dysfunction.

Understanding changes in taste perception in this patient population is crucial for developing targeted interventions to mitigate treatment-related side effects and improve post-treatment quality of life. However, there is limited literature that quantitatively evaluates taste perception before and after treatment in SCC patients, particularly in the context of tobacco exposure. As humans are widely recognized for their inherent inaccuracies in self-evaluating taste function [14], psychophysical testing provides a reliable and non-invasive method to assess gustatory function, enabling the measurement and the monitoring of taste changes over time [15].

This study aims to psychophysically evaluate taste perception in heavy-smoking patients undergoing glossectomy type IIIa for SCC of the tongue before and after treatment.

## Materials and methods

### Patient population

This prospective observational cohort study adhered to the principles outlined in the Declaration of Helsinki and received approval from the Ethics Committee for Clinical Experimentation of the University of Trieste. Written informed consent was obtained from all participants prior to their inclusion. Recruitment took place at the Trieste University Hospital, Trieste, Italy and at Treviso General Hospital, Treviso, Italy. Eligibility criteria required participants to be adults aged over 18 years with a confirmed diagnosis of SCC of the tongue, who were scheduled for glossectomy type IIIa (i.e. non-compartmental hemiglossectomy) according to Ansarin’s classification [16] and neck dissection, followed by adjuvant (chemo)radiotherapy as indicated by postoperative pathological findings. During the initial face-to-face patient interview, participants were asked to report a current heavy tobacco exposure, defined as a cumulative history exceeding 20 pack-years. Exclusion criteria included a prior history of head and neck malignancies, previous radiotherapy to the head and neck region, systemic diseases contraindicating surgical or adjuvant treatments, pre-existing diagnoses of taste disorders from other causes, being a candidate for total glossectomy due to the complete loss of tongue tissue precluding reliable psychophysical assessment of taste perception, or an inability to provide informed consent. Twenty no-smoker cancer-free controls were frequency matched to cases by age and sex, and were recruited among hospital staff and patients’ accompanying caregivers.

### Gustatory psychophysical evaluation

Gustatory evaluation was performed at baseline in both cases and controls using the validated taste strips test (Taste Strips, Burghart Messtechnik, Wedel, Germany) impregnated with four different concentrations of each of the tastes “sweet”, “sour”, “salty” and “bitter” performed strictly according to a standardised protocol [15]. The taste test is based on filter paper strips with a length of 8 cm and a tip area of 2 cm^2^ being impregnated with four different concentrations of each of the tastes “sweet” (strips A–D), “sour” (strips E–H), “salty” (strips I–L), and “bitter” (strips M–P). The first strips in each category (A, E, I, M) have the highest and the subsequent strips have lower taste concentration. For a standardized and reproducible performance of the taste test, the order of taste strips must be respected. At first, taste strips with low taste concentrations were presented. According to the increasing concentrations in each category, the strips with the highest concentrations were administered at the end of the test. Subjects were invited to move the strips from the left to the right side of the tongue. Participants had to identify the taste from a list of four descriptors: sweet, sour, salty, and bitter (multiple forced choice). At the end of the procedure, the taste strips score (TSS) was calculated (0–16 points).

### Follow-up evaluations

The gustatory psychophysical assessment was conducted again at 3, 6, 9, and 12 months after the surgical date for patients not undergoing adjuvant radiotherapy, and after the completion of adjuvant radiotherapy for those receiving it, following the same schedule for the control group and using the same methodology as at baseline. All patients were actively informed about the risks associated with persistent exposure to tobacco smoke and the importance of discontinuing exposure. During each follow-up evaluation, patients were asked about their smoking habits. Smoking cessation was defined as total abstinence from smoking, as reported consistently across all follow-up assessments. Patients who developed recurrences of the disease during follow-up were excluded from the study.

### Statistical analysis

Descriptive statistics were used to summarize the data for continuous variables, presented as mean ± standard deviation (SD), and for categorical variables, presented as absolute numbers and relative frequencies (percentages). Comparisons of continuous variables between groups of interest were performed using the Student’s t test for independent samples, while the Fisher exact test was applied for categorical variables. Two-way repeated-measures analysis of variance (ANOVA) was conducted to analyse TSS as a continuous variable. The factors considered were time (baseline, 3, 6, 9, and 12 months post-treatment), group (cancer patients and controls), and smoking status after treatment (continuers and quitters). The time-factor interactions were also investigated. Partial eta squared (η²) was calculated as a measure of effect size (strength of association) for each main effect and interaction in the ANOVA, with 0.01 to 0.059 representing a small effect, 0.06 to 0.139 a medium effect, and > 0.14 a large effect [17]. Post hoc analysis with Holm correction was performed for multiple comparisons in cases of significant ANOVA findings. The alpha level for statistical significance was set at 0.05, and statistical analysis was carried out using R software (version 4.2.3, 2023).

## Results

### Patient demographics

A summary of relevant demographic variables is provided in Table 1. The study cohort included 30 patients (mean [SD] age, 59 [14] years; 21 [70.0%] men) diagnosed with SCC of the tongue recruited between April 2022 and June 2024. According to inclusion criteria, all patients were heavy smokers, with a cumulative tobacco exposure exceeding 20 pack-years. Treatment included type IIIa glossectomy and ipsilateral neck dissection, including submandibular gland, for all patients. Reconstruction was achieved using pedunculated facial artery musculomucosal flap and anterolateral thigh flap in 11 (36.7%) and 19 (63.3%) cases, respectively; no patient received reinnervated reconstruction. Adjuvant radiotherapy was administered in 11 cases (36.7%). None of the patients received adjuvant chemoradiotherapy.

**Table 1:** Clinical characteristics

Characteristic	Heavy-smoker cases No. (%)	No-smoker controls No. (%)	*p*-value
No.	30	20	
Age, mean (SD), y	59 (14)	59 (9)	0.982
Sex			
Men	21 (70.0)	13 (65.0)	0.951
Women	9 (30.0)	7 (35.0)	
Alcohol status			
No drinker	16 (53.3)	15 (75.0)	0.148
Drinker	14 (46.7)	5 (25.0)	
Pathologic T category (AJCC-8)			
pT1	4 (13.3)		
pT2	9 (30.0)		
pT3	13 (43.3)		
pT4	4 (13.3)		
Pathologic N category (AJCC-8)			
pN0	17 (56.7)		
pN1	7 (23.3)		
pN2	6 (20.0)		
Reconstruction			
Free Flap	19 (63.3)		
Local Flap	11 (36.7)		
Adjuvant radiotherapy	11(36.7)		
Adjuvant chemoradiotherapy	0 (0.0)		

SD = Standard Deviation; y=years; AJCC= American Joint Committee on Cancer; T=tumor; N=node

### Taste perception: cases versus controls and changes over time

**Fig. 1:**
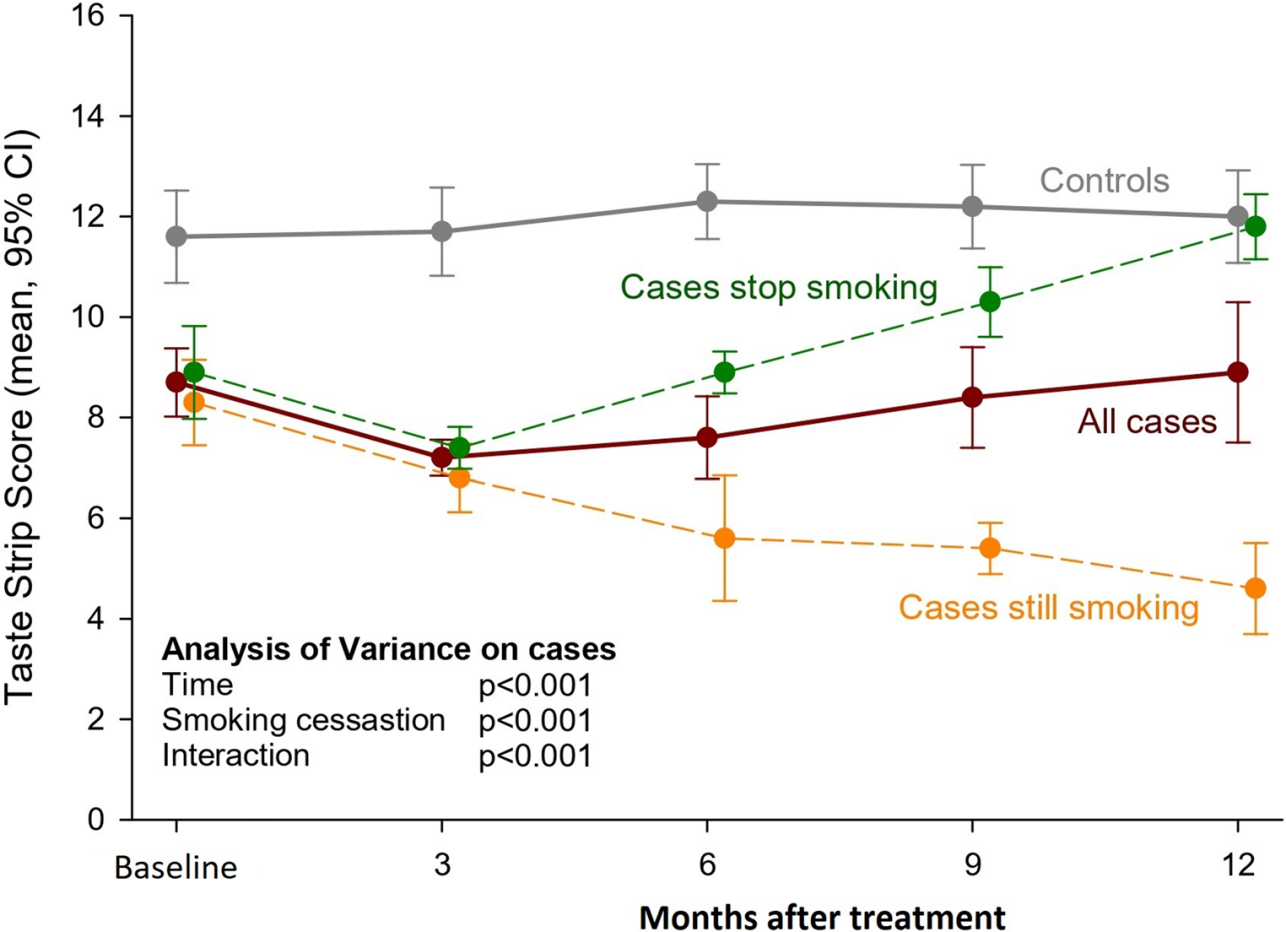
Evolution of gustatory function in cancer-free controls and patients, according to smoking habits CI: Confidence interval; TSS: Taste Strip Score

At baseline, cancer patients had notably lower mean TSS than controls (8.7 vs. 11.6, *p* <.001; Table 2), a trend that persisted throughout the follow-up period (Fig. 1). Following treatment, cancer patients exhibited a significant decline in taste perception at 3 months (mean [SD] TSS: 7.2 [1.0]), which was followed by a gradual recovery over subsequent evaluations at 6 (7.6 [2.3]), 9 (8.4 [2.8]), and 12 months (8.9 [3.9]). However, their TSS remained consistently lower than that of healthy controls across all time points (*p* <.01 for all comparisons).

**Table 2:** Mean (SD) of Taste Strip Score by groups and timing

	Mean (SD) TSS					
	Baseline	3 months after treatment	6 months after treatment	9 months after treatment	12 months after treatment	
Cases versus controls						
Cases (No.=30)	8.7 (1.9)	7.2 (1.0)	7.6 (2.3)	8.4 (2.8)	8.9 (3.9)	
Controls (No.=20)	11.6 (2.1)	11.7 (2.0)	12.3 (1.7)	12.2 (1.9)	12.0 (2.1)	
*p*-value	*p* <.001	*p* <.001	*p* <.001	*p* <.001	*p* =.002	
Cases by Reconstruction						
Free Flap (No.=19)	8.7 (1.8)	7.0 (1.05)	7.7 (1.7)	8.8 (2.6)	9.6 (3.9)	
Local Flap (No.=11)	8.5 (2.1)	7.5 (0.93)	7.4 (2.7)	7.6 (2.9)	7.7 (3.6)	
*p*-value	*p* =.792	*P* =.246	*p* =.714	*p* =.014	*p* =.212	
Cases by adjuvant RT						
Adjuvant RT (No.=11)	8.7 (2.1)	6.6 (1.0)	7.4 (2.9)	8.5 (2.5)	9.6 (4.3)	
No adjuvant RT (No.=19)	8.6 (1.8)	7.5 (0.9)	7.7 (1.8)	8.3 (2.9)	8.5 (3.7)	
*p*-value	*p* =.895	*p* =.027	*p* =.750	*p* =.893	*p* =.458	
Cases by smoking status after treatment						
Continuers (No.=12)	8.3 (1.5)	6.8 (1.2)	5.6 (2.2)	5.4 (0.9)	4.6 (1.6)	
Quitters (No.=18)	8.9 (2.0)	7.4 (0.9)	8.9 (0.9)	10.3 (1.5)	11.8 (1.4)	
*p*-value	*p* =.326	*p* =.147	*p* <.001	*p* <.001	*p* <.001	
Quitters versus Control						
Quitters (No.=18)	8.9 (2.0)	7.4 (0.9)	8.9 (0.9)	10.3 (1.5)	11.8 (1.4)	
Controls (No.=20)	11.6 (2.1)	11.7 (2.0)	12.3 (1.7)	12.2 (1.9)	12.0 (2.1)	
*p*-value	*p* <.001	*p* <.001	*p* <.001	*p* =.001	*p* =.0766	

SD = Standard Deviation; TSS = Taste Strip Score; RT = Radiotherapy

### Association between treatment modalities and taste outcomes

Subgroup analyses based on adjuvant treatment administration indicated that patients undergoing adjuvant radiotherapy had significantly lower mean [SD] TSS values at 3 months post-treatment compared to those who did not receive radiotherapy (6.6 [1.0] vs. 7.5 [0.9], *p* =.027). However, no significant differences were observed between the two groups at 6, 9, and 12 months post-treatment. Detailed values are reported in Table 2.

### Impact of smoking cessation on taste recovery

A subgroup of 12 patients (40.0%) continued smoking post-treatment, while 18 patients (60.0%) ceased smoking following their diagnosis. Patients in the two groups had similar taste level at baseline (Table 2), and no significant difference in mean [SD] TSS values between patients who ceased smoking (8.9 [2.0]) and those who continued smoking (8.3 [1.5]; *p* =.326). However, a divergent trend in taste emerged after 3 months from surgery (Fig. 1): patients who continued smoking exhibited a progressive deterioration in gustatory function over time, reaching the minimum 12 months after surgery (mean [SD] TSS, 4.6 [1.6]). Conversely, patients who quit smoking started recovering their gustatory ability, reaching a mean TSS level similar to controls 12 months after surgery (p for smoking cessation < 0.001). Repeated-measures ANOVA (Supplementary Table) revealed significant variation over time (*p* <.001), an effect of smoking cessation (*p* <.001), and a time-smoking-time interaction (*p* <.001).

## Discussion

The findings of this study highlight the significant impact of heavy smoking on taste perception in patients treated for SCC of the tongue. The effects of tongue resection on chemosensory function and patient perception have been previously investigated. Evidence indicates that taste disturbances are commonly reported following partial glossectomy with primary closure, suggesting that even limited surgical resections may significantly impact gustatory function and related quality-of-life domains [18]. Additional cross-sectional data combining psychophysical assessment with patient-reported outcomes have demonstrated a discrepancy between subjective and objective measures of taste function, potentially reflecting compensatory mechanisms such as retronasal olfaction [19]. In contrast to these studies, the present study adopts a longitudinal design in a surgically homogeneous cohort undergoing glossectomy type IIIa, with a specific focus on the role of smoking cessation as a modifiable determinant of gustatory recovery.

At baseline, patients with SCC of the tongue and heavy smoking habits exhibited marked gustatory dysfunction compared to non-smoker controls. This underscores the detrimental effects of prolonged tobacco exposure on the gustatory system, which likely exacerbates taste deficits associated with tongue cancer. SCC of the tongue can develop within the context of field cancerization, where clinically normal surrounding tissues exhibit pre-cancerous molecular changes [20, 21]. Recent findings highlight that the contralateral tongue tissue in patients with SCC shows distinct transcriptional and genomic abnormalities, including dysregulation of apoptosis-related genes [22]. These molecular signatures could not only facilitate cancer initiation but also contribute to taste dysfunction, reflecting the broader impact of field cancerization on tissue homeostasis.

The post-treatment trajectory revealed an initial decline in taste perception at three months, consistent with the effects of surgical intervention and adjuvant therapies, which can damage taste buds, salivary glands, and associated neural pathways. Encouragingly, the recovery of taste function was observed in subsequent follow-ups in patients who stopped smoking, while a progressive deterioration was reported in patients who continued to smoke. In the present series, 60% of patients stopped smoking after treatment of tongue SCC, a percentage comparable to what has been observed by other researchers when focusing on the subgroup of patients with smoking-related tumours [23]. Patients who abstained from smoking following their treatment experienced a significant recovery in taste perception over time, achieving a TSS comparable to those of the control group at 12 months. Conversely, patients who continued smoking exhibited a further progressive decline in gustatory function, indicating that persistent tobacco exposure not only impedes recovery but actively accelerates the deterioration of sensory capabilities. These findings highlight the critical importance of smoking cessation in promoting and optimizing post-treatment sensory recovery in this patient population.

In contrast, adjuvant radiotherapy seems to have a temporary effect on gustatory function, with pronounced changes evident in the short term. In fact, from the sixth month onward, taste function shows no significant differences between patients who received radiotherapy and those who did not. However, radiation therapy has the potential to induce long-term toxic effects, which may manifest beyond one year of follow-up [24]. Consequently, long-term evaluations are warranted to comprehensively assess the impact of radiation therapy on taste function.

Gustatory dysfunction has been associated with a reduced appetite, malnutrition, and a decline in quality of life. Notably, a low TSS has recently been identified as a predictive factor for unintentional weight loss [25]. This issue could be particularly important in patients with oral cancer, as gustatory dysfunction may exacerbate pre-existing malnutrition—a frequent condition in this population [26]. Pre-existing nutritional deficits often stem from the direct effects of the malignancy, its associated comorbidities, and the adverse outcomes of therapeutic interventions, which frequently impair swallowing function, further compromising their overall nutritional status [27].

Overall, this study underscores the need for targeted interventions to support smoking cessation and mitigate treatment-related taste deficits in patients with SCC of the tongue. Further research is warranted to explore the mechanisms underlying taste recovery and to develop tailored strategies for enhancing taste rehabilitation in this vulnerable patient group.

The study has several limitations that should be considered when interpreting its findings. The sample size is relatively small, with 30 patients and 20 healthy controls, which may limit the generalizability of the results and a comprehensive evaluation of covariates. However, to increase homogeneity, strict inclusion and exclusion criteria were applied. In addition, the assessment of tobacco exposure relies on self-reported data, raising the possibility that patients might underreport or misrepresent their smoking history. For this reason, we did not consider smoking reduction after treatment, which is an additional potential study limitation. Furthermore, while the taste strips test is a validated psychophysical method targeting the four primary tastes, it may not encompass the broader spectrum of oral sensory experiences, including the contribution of trigeminal sensitivity [28]. Although the exact resected volume was not recorded, the uniform adoption of glossectomy type IIIa across all cases minimizes inter-patient variability in the extent of surgical resection, thereby reducing its potential impact as a confounding factor. Patients were recruited between April 2022 and June 2024, during a period dominated by the SARS-CoV-2 Omicron variant, which has been associated with a substantially lower prevalence and severity of taste dysfunction compared to previous strains, as well as significantly faster recovery times [29, 30]. Thus, while no information on patient exposure to SARS-CoV-2 was collected, the potential impact on taste outcomes is presumed to be minimal, although this remains a limitation of the study. Finally, the study does not include a group of patients with tongue cancer who are non-smokers, which would have allowed for a better understanding of the specific impact of smoking on taste perception and recovery.

## Conclusion

In conclusion, this study highlights the detrimental effects of persistent smoking on taste perception in patients undergoing surgical treatment for SCC of the tongue. Smoking cessation plays a pivotal role in promoting the recovery of gustatory function, while continued smoking further impairs taste after treatment. These findings emphasize the critical need for integrating smoking cessation programs into the treatment plan to enhance gustatory function recovery.

## Supplementary Information

Below is the link to the electronic supplementary material.

Supplementary file1 (DOCX 16.6 KB)

## Data Availability

The authors confirm that the data supporting the findings of this study are available within the article.
