# Supplementary material for: Effects of smoking cessation on taste function in heavy smokers undergoing hemiglossectomy for tongue squamous cell carcinoma
Source: Eur Arch Otorhinolaryngol. 2025 Jul 23;282(10):5315–21. doi: 10.1007/s00405-025-09590-8 (PMC12518390; doi:10.1007/s00405-025-09590-8)
Supplement: Supplementary file 1 — Supplementary file1 (DOCX 16.6 KB) [file 405_2025_9590_MOESM1_ESM.docx]

**Supplementary Table. Results of the two-way repeated measures ANOVA for Taste Strip Score**

|  | Two-way repeated measures ANOVA  Factor: Group (cases versus controls) | | | Two-way repeated measures ANOVA  Factor: Post-treatment smoking status (continuers versus quitters) | | |
| --- | --- | --- | --- | --- | --- | --- |
|  | Time | Group | Time*Group | Time | Smoke | Time*Smoke |
| F-value | 3.01 | 50.4 | 3.5 | 6.6 | 103.6 | 33.6 |
| p-value | 0.019 | <0.001 | 0.009 | <0.001 | <0.001 | <0.001 |
| Partial eta squared ($\boldsymbol{\eta}^{\mathbf{2}}\mathbf{)}$* | 0.06 | 0.07 | 0.51 | 0.19 | 0.79 | 0.55 |

* Interpretation of η2: η2 = 0.01, η2 = 0.06 and η2 = 0.14 indicate respectively a small, medium and large effect
